# Tranexamic Acid Use for Massive Hemoptysis in a Child: A Case Report

**DOI:** 10.7759/cureus.28186

**Published:** 2022-08-19

**Authors:** Ahlam Mazi

**Affiliations:** 1 Department of Pediatrics, King Abdulaziz University, Jeddah, SAU

**Keywords:** non-cystic fibrosis bronchiectasis, tranexamic acid, pulmonary hemorrhage, massive hemoptysis, hemoptysis

## Abstract

Massive hemoptysis is a rare life-threatening condition in children. Individuals with non-cystic fibrosis bronchiectasis may present with various degrees of hemoptysis. Therapeutic measures are mainly derived from studies involving adults or various case reports of children with cystic fibrosis. The standard management of massive hemoptysis is limited to invasive bronchoscopy, bronchial artery embolization, and surgical resection. Tranexamic acid (TXA) use is limited to non-massive hemoptysis or as an adjuvant and temporizing measure before definitive treatment. We report the potential use of TXA as an emergency treatment for massive hemoptysis in a 10-year-old boy with non-cystic fibrosis bronchiectasis and chronic infection. The use of systemic TXA (250 mg every eight hours for five days) successfully stopped active bleeding beginning from the first dose and altered the need for invasive interventions. Although he experienced another episode of massive hemoptysis because of pneumonia and pulmonary exacerbation, invasive measures were not required because he responded to systemic TXA immediately. Moreover, no further recurrence of hemoptysis was noted on cessation of TXA and throughout two years of regular follow-up. Therefore, TXA could be considered a non-invasive therapy for children with massive hemoptysis, especially in the absence of standard invasive therapies.

## Introduction

Hemoptysis is a rare and potentially life-threatening condition with various causes. Pneumonia is the leading cause of hemoptysis in children [1-3]. Up to 16% of children with non-cystic fibrosis bronchiectasis may have hemoptysis [4]. Quantification of the volume of expectorated blood is crucial for assessing the severity of hemoptysis. Life-threatening hemoptysis in children is defined as a net loss of >8 mL/kg or >200 mL of blood within 24 hours and is associated with high mortality without proper therapeutic intervention [2,5,6]. High-pressure bronchial artery circulation is the source of active bleeding in 90% of massive hemoptysis cases [7]. It originates in the thoracic aorta and intercostal arteries; however, other variations may exist. The standard therapeutic options for massive hemoptysis are interventional bronchoscopy with topical airway vasoconstrictors, laser bronchoscopy, balloon tamponade, bronchial artery embolization (BAE), and surgical lobectomy [5]. Tranexamic acid (TXA) is a synthetic antifibrinolytic agent that has been used for adults to decrease the severity of non-massive hemoptysis. It is also considered an adjuvant therapy for refractory hemoptysis [7-12]. Our case report addresses the possible use of TXA as a first-line treatment for massive hemoptysis in a child with non-cystic fibrosis bronchiectasis.

## Case presentation

A 10-year-old boy with ataxia telangiectasia presented to the emergency department with a one-month history of worsening wet cough and two-day history of hemoptysis. The last episode of hemoptysis occurred one hour before presentation, when he expectorated ≥250 mL.

He had no history of fever, epistaxis, hematemesis, bruises, trauma, breathing difficulty, dyspnea, shortness of breath, or chest pain. Previously, he had recurrent chest infections (three episodes per year); some of those infections required admission for intravenous antibiotics.

**Figure 1:**
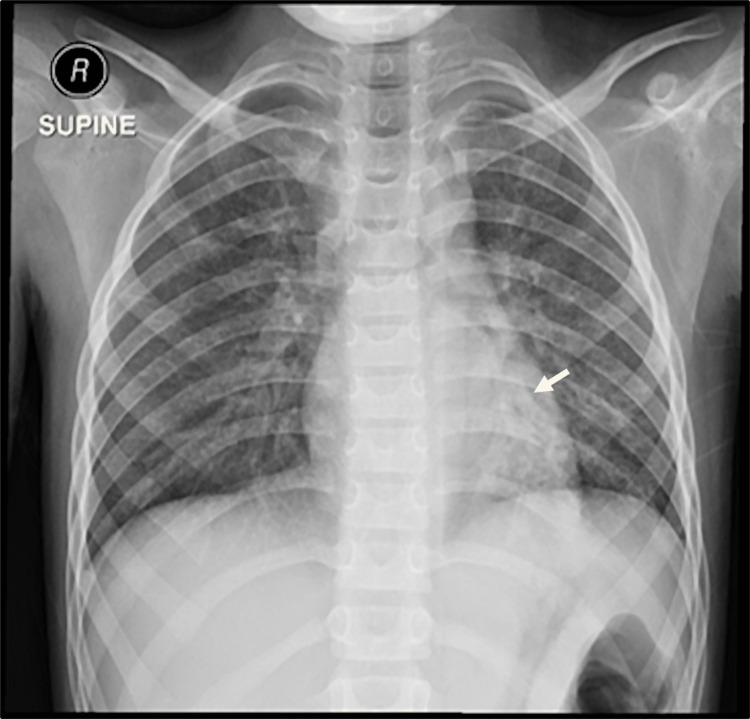
Chest X-ray The chest X-ray evaluation showed airspace opacity over the left lower lobe.

His parents were first-degree cousins and his younger sister was recently diagnosed with ataxia telangiectasia. Initially, the patient was hemodynamically stable, with a normal respiratory rate (20 bpm) and oxygen saturation (97% SpO2) on room air. He had oculocutaneous telangiectasia, nystagmus, ataxic gait, and significant muscle wasting in both the upper and lower limbs. He used a wheelchair but was able to walk a few steps with support. He had bilateral crackles with decreased air entry over the left lower lung area. Further investigation showed leukocytosis (white blood cell count 15.6×109/L) with neutrophilia (64%), a hemoglobin level of 13 g/dL, and a platelet count of 617×109/L; his coagulation profile and inflammatory markers were normal. Respiratory virus polymerase chain reaction test, Mycobacterial tuberculosis polymerase chain reaction, acid-fast staining, and culture test results were negative. A chest radiograph showed left lower lung opacity (Figure 1). An upper airway assessment and upper gastrointestinal tract endoscopy yielded normal results.

**Figure 2:**
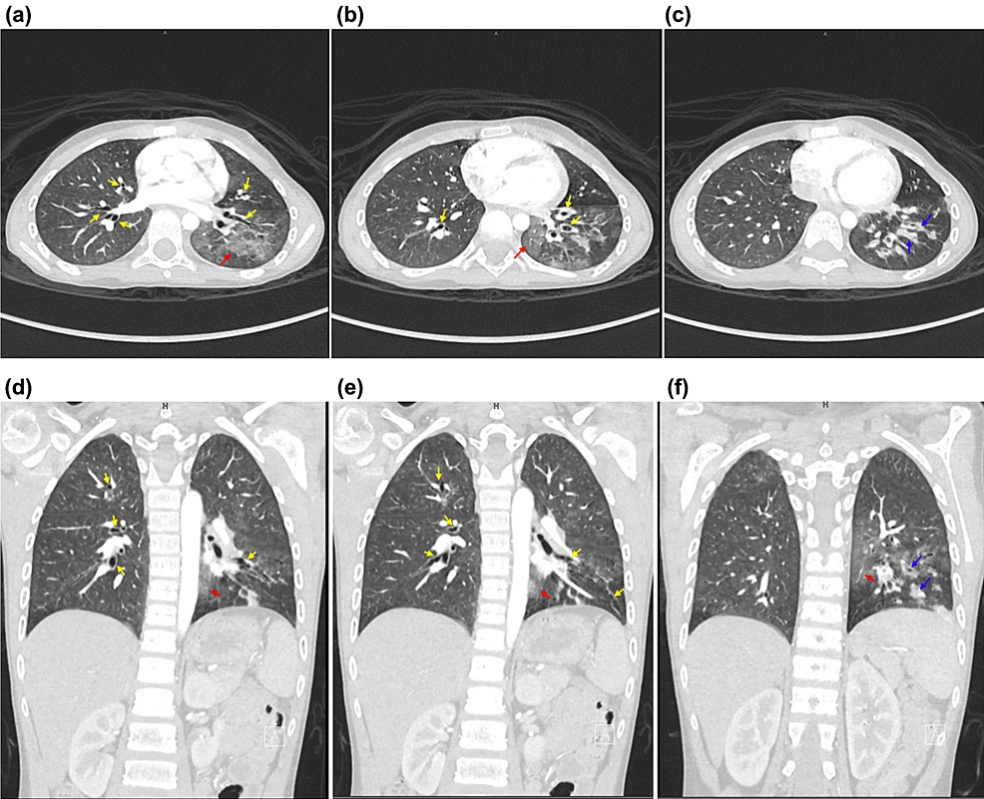
Computed tomography angiography Computed tomography (CT) showed bilateral ground-glass air-space opacity on the left side and an area of enhancement over the left lower lobe (red arrows). Bilateral cylindrical bronchiectasis affected all the lobes (yellow arrows). However, this was more prominent in the left lower lobe. Thickening of the bronchial wall was observed over the left lower lobe, and some contained fluid components (blue arrows). Three bronchial arteries were observed: one branch on the right side and two left bronchial arteries measuring 0.2 cm. Tortuosity or extravasation was not observed. Bilateral dilatation of the pulmonary arteries and bilateral hilar lymph node enlargement were observed. The largest lymph node was on the left and measured 1.5 cm. The cardiomediastinal structure and major vessels were unremarkable.

Intravenous piperacillin-tazobactam and vancomycin were initiated as treatments for pneumonia. Thereafter, he experienced two episodes of massive hemoptysis associated with melena. He became hemodynamically unstable and experienced a significant decrease in the hemoglobin level (10 g/dL) that required non-invasive respiratory support, ringer lactate, fresh-frozen plasma, and packed red blood cell transfusion. Computed tomography angiography showed bilateral ground-glass opacity, mainly in the left lower lobe, suggestive of pulmonary hemorrhage (Figure 2). Rigid bronchoscopy revealed pooling of blood with clots in the left tertiary bronchi and blood clots in the left main bronchus. Because the standard treatment for massive hemoptysis and BAE were not available, intravenous TXA 250 mg every eight hours was administered for five days. The patient was closely monitored for thromboembolic events, seizures, or hypersensitivity reactions which are potential adverse effects of TXA. No further episodes of hemoptysis were noted. When the patient was stable, monthly intravenous immunoglobulin, inhaled budesonide, prophylactic azithromycin, and airway clearance treatment with hypertonic saline and chest physiotherapy were started.

After a few months, he experienced a similar episode of massive hemoptysis associated with fever and worsening cough for several days. He was pale, ill, and had respiratory distress requiring oxygen (non-rebreather mask) associated with a significant decrease in hemoglobin (10 g/dL) requiring fresh-frozen plasma and packed red blood cell transfusions. Because of our limited resources, and in the context of a previous response to medical treatment, intravenous TXA 250 mg every eight hours was started immediately as a treatment for massive hemoptysis. At the time of admission, the patient did not experience any further episodes of hemoptysis or require invasive intervention. The patient continued to receive monthly intravenous immunoglobulin, daily inhaled budesonide, prophylactic azithromycin, and airway clearance with chest physiotherapy and nebulized hypertonic saline. Thereafter, no further episodes of severe respiratory exacerbation or hemoptysis occurred during two years of regular follow-up. Our patient tolerated systemic TXA without any serious or minor side effects.

## Discussion

Hemoptysis is the expectoration of blood from the lower respiratory tract. It is uncommon in children. The majority of cases present as self-limited non-massive hemoptysis that does not require invasive therapies. Infectious causes, including pneumonia, bronchitis, bronchiectasis, and pulmonary tuberculosis, are the most common. However, other unique causes of hemoptysis in children include foreign body aspiration, cystic fibrosis, and congenital heart diseases [1-3]. For children, massive hemoptysis is defined as a net loss of >8 mL/kg or >200 mL of blood within 24 hours [2,5]. Although it is rare, it is a life-threatening condition associated with high mortality (>50%) caused by asphyxia, respiratory distress, and hemodynamic instability [6]. Therefore, establishing the cause and assessing the severity of hemoptysis are crucial for the management of massive hemoptysis.

The lung has dual circulation: high-volume, low-pressure pulmonary arterial circulation and low-volume, high-pressure bronchial circulation. In most cases, the latter originates from the thoracic aorta and intercostal arteries. It mainly perfuses the conducting airways to the level of the terminal bronchioles. Chronic and recurrent infections and inflammation damage the airways and bronchial arteries. Therefore, bronchial arteries become tortuous and dilated with an increase in blood flow. Additionally, chronic inflammation stimulates neovascularization and angiogenesis of collateral thin-walled fragile vessels that originate from systemic circulation. Massive hemoptysis is usually caused by active bleeding of the high-pressure circulation in 90% of cases [7].

Massive hemoptysis management aims to stabilize the airway and control active bleeding. Standard therapies for massive hemoptysis include bronchoscopy, BAE, and surgical lobectomy [5,7]. Bronchoscopy can be used to identify the site of bleeding and facilitate various techniques to achieve hemostasis. Although both flexible and rigid bronchoscopy can be used, rigid bronchoscopy provides better airway control and allows the rapid removal of clots and debris. To control active bleeding during bronchoscopy, topical epinephrine, ice saline, fibrinogen-thrombin glue, and Bortopase were used. Other measures include carbon dioxide or Nd-YAG laser and balloon tamponade of the bronchial lobe or the main bronchus using a Fogarty catheter. Selective BAE is an effective but invasive intervention that is considered for refractory cases or failure to localize the bleeding site via a bronchoscope. The bleeding vessel was identified using bronchial arteriography or computed tomography angiography. Thereafter, selective embolization reduces perfusion pressure to the affected bronchial artery. Several studies have shown that BAE is successful in more than 90% of cases. However, recurrence of hemoptysis was noted in 20% to 40% of cases that required further BAE [5,13,14]. Most complications after BAE are minor and self-limiting. Rarely do serious complications occur and cause neurological impairment attributable to embolization of the spinal artery [5].

Surgical resection is usually considered a second-line treatment for refractory cases despite multiple BAE procedures or the failure of all previous measures. Segmentectomy, lobectomy, and pneumonectomy have been reported and are dependent on the underlying pathology. Emergent surgical resection for massive hemoptysis has a mortality rate of 35% to 40%; however, elective surgical resection has a mortality rate of 0% to 18% [15,16].

TXA is a synthetic analog of lysine that inhibits the conversion of plasminogen into active plasmin. Therefore, it prevents fibrin degradation and stabilizes clot formation. It is used to control active bleeding in various disorders. It can be administered intravenously, orally, or nebulization depending on the site and the severity of active bleeding. When administered, patients should be monitored for potential adverse effects which include hypersensitivity reactions, thromboembolic events, hypotension, nausea, vomiting, headache, and seizures. It should not be used in patients with hypersensitivity to tranexamic acid, hypercoagulopathy, subarachnoid hemorrhage, active thromboembolic disease, or a history of a thromboembolic event, or at risk for thrombosis or thromboembolism [17]. Bellam et al. found that the use of TXA for patients with submassive hemoptysis decreased the severity of hemoptysis and could be used as a temporary measure before definitive treatment [8]. Similarly, a systematic review showed that TXA reduced the bleeding time and frequency of hemoptysis; however, it had no effect on pulmonary hemorrhage remission. Therefore, there is insufficient evidence to support its use as a definitive treatment [18]. Additionally, the use of TXA significantly decreased the hospital admission rate (50%) for adult cystic fibrosis patients with recurrent hemoptysis. However, there was no change in the frequency of respiratory exacerbations, the incidence of hemoptysis, or severity [9]. Moreover, TXA was used as an adjuvant therapy for refractory hemoptysis in children with cystic fibrosis who underwent multiple BAE procedures; however, there is no clear evidence of remission with TXA [11,12]. O’Neil et al. concluded that inhaled TXA is safe and effective for critically ill children with pulmonary hemorrhage. Cessation of bleeding was observed within 48 hours in most cases. However, the majority of the study population (47%) had a diffuse alveolar hemorrhage or bleeding related to aortopulmonary collateral arteries in patients with congenital heart disease (21%) [19]. Therefore, TXA has never been used as a definitive treatment for massive pulmonary hemorrhage in children with non-cystic fibrosis bronchiectasis or other infectious causes.

Ataxia telangiectasia is a multisystemic disorder caused by mutations in the ATM gene. Affected individuals have progressive pulmonary disease, neuromuscular weakness, and immunodeficiency, and they are at increased risk for malignancy. Pulmonary complications are not uncommon for affected subjects; in fact, they are major causes of morbidity and mortality. Individuals have chronic pulmonary infections and inflammation caused by recurrent pulmonary infections, poor airway clearance, dysphagia, aspiration, and immunodeficiency. Ultimately, this vicious cycle of chronic infection and inflammation leads to bronchiectasis, interstitial lung disease, and pulmonary fibrosis [20]. Our patient presented with massive hemoptysis secondary to chronic infection and bronchiectasis. Although it is not a common presentation, it has been reported that 16% of patients with non-cystic fibrosis bronchiectasis may present with various degrees of hemoptysis [4]. Unfortunately, the patient continued to experience massive pulmonary hemorrhage despite undergoing interventional bronchoscopy. For these cases, BAE is the standard therapeutic option. Because of limited resources, emergency treatment using TXA has been proven to be beneficial for stopping massive hemoptysis. Subsequently, no further episodes of hemoptysis were noted after TXA discontinuation. This is different from what was previously reported regarding patients with cystic fibrosis [12]. Although the patient experienced relapse a few months later in the context of respiratory exacerbation, he continued to respond to TXA. Neither bronchoscopy nor BAE was required and no subsequent events of massive hemoptysis occurred during two years of regular follow-up.

This case report addresses the potential benefits of TXA as a first-line treatment for massive hemoptysis in children with non-cystic fibrosis bronchiectasis. However, further studies are required to assess the safety and efficacy of TXA as first-line therapy for massive hemoptysis in children.

## Conclusions

Massive hemoptysis is a rare, life-threatening condition with a high mortality rate if left untreated. The established standards of care are limited to invasive bronchoscopy, BAE, and surgical resection. The use of TXA has been limited to non-massive hemoptysis or as an adjuvant or temporary measure before definitive treatment. Our case report addresses the potential benefits of TXA as a therapeutic measure for acute massive hemoptysis. TXA can successfully treat massive hemoptysis in patients with bronchiectasis, thus altering the need for invasive therapeutic intervention. However, further prospective studies are required to establish the safety and efficacy of TXA as a first-line treatment for massive hemoptysis in children.
